# Minimalist Training: Is Lower Dosage or Intensity Resistance Training Effective to Improve Physical Fitness? A Narrative Review

**DOI:** 10.1007/s40279-023-01949-3

**Published:** 2023-11-04

**Authors:** David G. Behm, Urs Granacher, Konstantin Warneke, Jose Carlos Aragão-Santos, Marzo Edir Da Silva-Grigoletto, Andreas Konrad

**Affiliations:** 1 School of Human Kinetics and Recreation, Memorial University of Newfoundland, St. John’s, Newfoundland and Labrador A1C 5S7 Canada; 2 Department of Sport and Sport Science Exercise and Human Movement Science, University of Freiburg, Freiburg, Germany; 3 Institute for Exercise, Sport and Health, Leuphana University, Lüneburg, Germany; 4 Department of Physical Education, Post Graduate Program in Health Sciences, Federal University of Sergipe, São Cristóvão, Brazil; 5 Institute of Human Movement Science, Sport and Health, Graz University, Graz, Austria

## Abstract

**Background:**

Findings from original research, systematic reviews, and meta-analyses have demonstrated the effectiveness of resistance training (RT) on markers of performance and health. However, the literature is inconsistent with regards to the dosage effects (frequency, intensity, time, type) of RT to maximize training-induced improvements. This is most likely due to moderating factors such as age, sex, and training status. Moreover, individuals with limited time to exercise or who lack motivation to perform RT are interested in the least amount of RT to improve physical fitness.

**Objectives:**

The objective of this review was to investigate and identify lower than typically recommended RT dosages (i.e., shorter durations, lower volumes, and intensity activities) that can improve fitness components such as muscle strength and endurance for sedentary individuals or beginners not meeting the minimal recommendation of exercise.

**Methods:**

Due to the broad research question involving different RT types, cohorts, and outcome measures (i.e., high heterogeneity), a narrative review was selected instead of a systematic meta-analysis approach.

**Results:**

It seems that one weekly RT session is sufficient to induce strength gains in RT beginners with < 3 sets and loads below 50% of one-repetition maximum (1RM). With regards to the number of repetitions, the literature is controversial and some authors report that repetition to failure is key to achieve optimal adaptations, while other authors report similar adaptations with fewer repetitions. Additionally, higher intensity or heavier loads tend to provide superior results. With regards to the RT type, multi-joint exercises induce similar or even larger effects than single-joint exercises.

**Conclusion:**

The least amount of RT that can be performed to improve physical fitness for beginners for at least the first 12 weeks is one weekly session at intensities below 50% 1RM, with < 3 sets per multi-joint exercise.

## Key Points

**Table:** 

In the first 8–12 weeks, resistance training-hesitant individuals should begin with a single progressive resistance training session per week with at least one set of 6–15 repetitions ranging from 30 to 80% of one-repetition maximum using multi-joint functional movements.
Very low intensity activities such as prolonged static stretching (> 10 min of static stretching per muscle group) can improve strength and hypertrophy albeit with much longer duration training sessions.
It is unknown if these minimalist training recommendations would still be effective after 8–12 weeks of resistance training and thus it may be necessary to provide progressively greater frequencies, volumes, and intensities of training.

## Introduction

Resistance training (RT) was not included in early (e.g., American College of Sports Medicine (ACSM) 1978) position statement recommendations and prescriptions for activity to ensure health in adults [1, 2]. In addition to the highly cited benefits of RT for muscle strength, hypertrophy, and endurance [3–7], the health benefits of RT are now well established. RT has been positively associated with reducing cardiovascular risk factors such as reductions in blood lipids (i.e., total cholesterol, low-density lipoprotein cholesterol concentrations, and triglycerides), blood pressure, obesity, and glucose intolerance [8–12]. These RT-induced responses result in fewer reported incidences of diabetes, stroke, cancer, dementia, arthritis, coronary artery disease, and pulmonary disorders [8–10, 13, 14]. Evidence from a recent meta-analysis [15] demonstrated that muscle strengthening (i.e., RT) activities reduce the relative risk of all-cause mortality, cardiovascular disease, total cancer, and diabetes by 10–17%. Another meta-analysis reported that RT was associated with 21% and 40% lower all-cause mortality rates alone and when combined with aerobic exercise, respectively, when compared with no exercise [16]. Depending on the health outcome under investigation (e.g., insulin resistance, hypertension, heart rate variability, cardiovascular and cardiometabolic syndrome), between 30 and 60 min of weekly RT have the largest effect [3–10]. Circuit-based RT [17] and blood flow restriction with RT [18] has been shown to improve maximum oxygen uptake (3.8–13.4%). To achieve large magnitude improvements in maximum oxygen uptake, the authors recommended 14–30 sessions for 6–12 weeks, with each session lasting at least 20–30 min, at intensities between 60 and 90% of one-repetition maximum (1RM) [17]. RT has also been shown to improve body composition, skeletal bone health, independent living, and mobility in older adults, decreasing back pain, falls, and fractures, and contributing to fewer functional (multi-joint movements mimicking daily tasks) limitations [10, 13, 14, 19, 20]. Impaired balance, which can lead to falls resulting in fractures, especially with seniors, can also be enhanced with traditional RT exercises as well as RT exercises performed on unstable surfaces [21, 22]. Seniors can again benefit, as RT can contribute to improved cognitive function and memory performance. Apparently healthy individuals and individuals with psychological symptoms can experience increased feelings of well-being, self-efficacy, and reductions in schizophrenia, anxiety, depression, and other mood disorders with RT [10, 11, 23, 24].

With such an important health-promoting activity, every individual might want to include RT among other health strategies (i.e., aerobic exercise, proper nutrition, among others) in their activities of daily living. However, this is not the case. In Canada 25% of adults aged 18–79 years participate in the recommended RT at least twice per week [25] (Statistics Canada, Canadian Health Measures Survey Activity monitor data 2018–2019]). US statistics are similar, with a survey of 397,423 US citizens showing that only 30% of American adults engage in muscle strengthening activities and 24% meeting recommended aerobic and muscle strength activity guidelines (2 days/week) [26]. In Australia, in a survey of 195,926 participants, aged 15–98 years, only 10.4% and 9.3% met the muscle-strengthening activity recommendations (two times/week, 150 weekly minutes of moderate-vigorous intensity activity) over 2 weeks and within a year, respectively [27]. Similarly, a cross-sectional study of English adults (*n* = 253,423; 18- to 65-year-olds) found that 29% of men and 24% of women met the guidelines for the Health Survey for England definition of strengthening activity, whereas 16% of men and 9% of women met the strengthening guidelines for which evidence of health-related benefits could be found; furthermore, using the most-stringent definition in UK physical activity guidelines (two times/week, 150 weekly minutes of moderate-intensity activity), only 7.3% of males and 4.1% of females achieved the recommendations for strengthening activities [28]. In both the Australian and the English surveys, older individuals (50+ years), and individuals in socioeconomically disadvantaged or deprived areas or rural areas and those with lower education were less likely to report sufficient muscle-strengthening activities [26, 28]. Low participation rates were also reported by Garcia-Hermoso et al. [29] when reviewing the World Health Organization (WHO) guidelines for aerobic and muscle strengthening activities including over 3 million individuals from 31 countries. Garcia-Hermoso et al. [29] reported that adherence to these guidelines was 17.1%, 13.6%, and 19.5% in young and middle-aged adults, older adults, and adolescents, respectively. The WHO guidelines for adults and older adults recommend two or more days a week of muscle-strengthening activities at moderate or greater intensity. Recommendations for children and adolescents are for muscle-strengthening activities at least 3 days per week [30].

The low participation rates for RT have been attributed to a number of factors. Higher intensity exercise can contribute to the exercise apathy [31]. Many people do not have positive experiences with exercise, since their physiological responses to exercise associated with neurotransmitters (e.g., dopamine, and serotonin associated with cortical reward centres) [32, 33], endorphin or enkephalin (morphine-like hormones that inhibit pain and increase feelings of well-being) [34, 35] release are muted compared to others who enjoy exercise. Hence, there is little or no reward of a runner’s or exercise “high” (e.g., drug-induced euphoria) to provide exercise enjoyment and motivation to continue. Diurnal rhythms play a role in the motivation to exercise with the majority of people more motivated to move in the afternoon (≥ 15:00) [36]. Hence, for individuals with lower motivation for exercise, a morning training program would be even more problematic. Another common problem is the lack of time available for exercise [37–39]. According to a narrative review by Fyfe et al. [40], since adherence to traditional RT is poor, “minimal-dose RT involving lower session volumes with either (1) higher training intensities/loads performed at lower frequencies or (2) lower training intensities/loads performed at higher frequencies and with minimal-to-no equipment may be more feasible approaches to improving muscle strength and function across the lifespan.”

While most researchers, coaches, and athletes want to know the optimal exercise prescription for the greatest fitness component gains (i.e., aerobic capacity, muscle strength, endurance, power, flexibility), most sedentary individuals want the minimum recommendations for fitness and health benefits [1]. Haskell proposed a theoretical dose–response curve that illustrated the greatest benefits occur with individuals with the lowest baseline activity (sedentary), and this activity/benefits relationship plateaus for high baseline activity individuals (active or trained) [1].

Thus, the objective of this narrative review was to investigate and identify lower than typically recommended RT dosages (i.e., shorter durations, lower volumes, and intensity activities) that can improve fitness components such as muscle strength, hypertrophy, and endurance for sedentary individuals or beginners not meeting the minimal recommendation of exercise. Since increased muscle strength and endurance are associated with improved health outcomes and mortality [41–45], identifying minimal RT recommendations should provide benefits to both physical performance (e.g., muscle strength, endurance, power, and hypertrophy) [7, 46–49] and health. A narrative review rather than a systematic meta-analysis was chosen since the high heterogeneity of the study protocols (e.g., different RT frequencies, sets, repetitions, intensities, types of exercise) and measures (e.g., isometric, isoinertial, and isokinetic strength, isokinetic and jump power, muscle endurance (duration, number of repetitions)) would not allow for an evaluation or comparison of “similar” studies.

## Resistance Training Frequency

Twelve years following the original ACSM position statement that excluded RT recommendations [2], the 1990 ACSM position stand recommended one set of 8–12, moderate-intensity RT exercises at least two times per week per muscle group [50]. This recommendation is in accord with other reviews and association recommendations, which provide typical minimum recommendation for RT weekly frequency of two to three times per week in order to achieve significant increases in muscle strength, endurance, and hypertrophy [19, 25, 26, 30, 49, 51, 52]. Multiple weekly RT sessions are often reported to provide greater positive strength and endurance adaptations than fewer sessions; 4 > 2 [53], 3 > 1 [54], 5 > 1 [55, 56], which would suggest that greater RT volumes may be more effective. However, the literature is not unanimous. College-aged men who had previously done RT for at least 6 months, performed powerlifting (bench press, squats, and deadlifts) RT for 6 weeks, and found that 6 days per week did not provide greater strength or hypertrophy gains than training 3 days per week when volume and intensity were equated [57]. Lasevicius et al. [58] reported that RT men who had previously trained 3 days per week for at least 1 year reported that 2 days per week RT produced similar increases in muscular adaptations as 3 days per week; however, effect size magnitudes for muscle hypertrophy were greater when training 2 versus 3 days. A meta-analysis by Cuthbert et al. [59] found that over a 6- to 12-week training period, there were no significant differences in strength development between training frequencies (one to nine times per week) when training volumes were equated in well-trained populations. Hence, if well-trained RT individuals can benefit from lower training frequencies, it would be suspected that lesser trained individuals would also receive significant benefits even with a lower frequency RT schedule. A meta-analysis reported that untrained individuals experience their greatest gains with RT 3 days per week at 60% 1RM, while trained individuals show more optimal gains with 2 days per week at 80% 1RM [60]. The higher volumes and intensity of RT per session with the trained individuals may account for the need for fewer training sessions per week in order to provide more time for overcompensation adaptations. Hence, multiple RT sessions per week are recommended and provide meaningful gains [61, 62], but more is not always significantly more beneficial than less (e.g., 6 vs. 3, 3 vs. 2) [57, 58, 61].

There are a number of studies demonstrating that significant strength gains can even be achieved with a single training session per week. For instance, Brigatto et al. [63] reported no significant differences in muscle strength and endurance when comparing one versus two RT sessions per week equated for training volume over 8 weeks with RT young men (4.1 ± 1.8 years of RT). Following 8 weeks of RT, untrained young adult males displayed no significant differences in muscle strength and hypertrophy when training was reduced to either one- or two-volume equated training session days per week in untrained men [64]. Similar results were reported by Graves et al. [65] who had adult participants (24 men and 26 women) experience 12 weeks of reduced training volume after either 10 or 18 weeks of RT. They found no significant muscle strength differences when reducing training to 1 or 2 days per week. In the same vein, but with pubescent male baseball players, DeRenne et al. [66] reported no significant difference in leg press or pull-up strength measures when 12 weeks of in-season maintenance training was reduced to 1 or 2 days per week following 12 weeks of RT. A meta-analysis by Ralston et al. [62], while reporting greater strength gains with moderate and high weekly set volumes, still found large magnitude effect size gains with single sets in novice and intermediate male trainees.

Faigenbaum et al. [67] also demonstrated no significant differences in children 7–12 years of age, between RT 1 or 2 days per week over 8 weeks. In another 8-week, volume-equated, RT program (Nordic hamstrings exercise) for youth soccer players, no significant differences were detected between the 1- and 2-day RT groups; however, three of four tests (40-m sprint, change of direction, standing long jump) exhibited larger magnitude effect sizes with the twice-weekly group [68].

For sedentary or less active individuals, lower prescribed RT frequencies may be sufficient to induce muscular adaptations, which is in line with the law of diminishing returns [69]. If the recommended training frequency for trained or active individuals is too high for the sedentary individual, then overtraining will occur that could result in diminished physical adaptations. Hence, for the previously sedentary individual, multiple RT sessions per week may not provide additional benefits and, thus, without proportional improvements for the additional effort, the enjoyment, motivation, or enthusiasm for exercise may diminish [69]. Although there is some disagreement [59], if optimal or more substantial muscle strength, endurance, and hypertrophy gains are the goal, then multiple weekly training sessions are recommended over single weekly sessions, especially for the more highly trained or active individuals [19, 25, 26, 30, 49, 51–56]. However, the evidence does indicate that a RT program beginning with a single session per week can provide strength gains for sedentary or less active individuals who are only interested in the minimal weekly RT frequency to attain significant muscle strength and endurance gains over at least 8–12 weeks. The duration of improvements with single weekly RT sessions may persist for longer than 8–12 weeks, but there is little to no research investigating single RT sessions per week for more than 3 months. If a RT plateau is reached after 12 weeks, an increase in training frequency would be recommended.

## Resistance Training Volume

### Sets

The appropriate number of sets to obtain optimal muscle strength and endurance adaptations was investigated soon after World War II, when Delorme recommended the now ubiquitous prescription of three sets of 8–12 repetitions [70, 71]. There are many articles demonstrating the superiority of three sets of RT over one set with RT programs of six [72–74], nine [75], or 12 weeks [76]. While it seems that < 3 sets could still be effective in RT beginners, > 3 to 4 sets of training have been reported to result in a ceiling effect with, for example, older adults [52]. There may also be a muscle-specific effect with a lower number of sets (i.e., single set) providing superior training adaptations with upper body training versus more pronounced training gains with three sets versus one set for the lower body [72]. For optimal muscle hypertrophy, Dankel et al. [77] suggested in their review that with trained individuals, similar muscle groups should be trained more frequently while reducing the number of sets per training session. They indicated that increasing set number past a certain threshold or ceiling has negligible effects on muscle hypertrophy.

Untrained women who participated in a 24-week RT program demonstrated that one set provided similar strength gains as two to four sets for the first 12 weeks, but the multiple sets were more effective over the full 24-week period [78]. As mentioned in the previous section, with training doses that are too high, overtraining and diminished training returns may occur. In accordance with this rationale, Rhea and colleagues’ [60] meta-analysis illustrated a strength development dose–response relationship following RT, with maximal strength gains in both trained and untrained individuals with four sets per muscle group. Five and six sets resulted in diminished strength gains. Peterson et al.’s [79] meta-analysis differentiated between trained states and summarized that the optimal number of sets for large effect size magnitude strength gains for untrained individuals was approximately three sets, whereas for trained and athletic individuals, large magnitude improvements would be experienced with four and eight sets, respectively. However, while three sets could provide very large magnitude effect size ratio increases over 2.0 for the untrained, one set still induced large magnitude strength gains in the previously untrained, with effect sizes exceeding 1.0. From this review of the literature, it is difficult to find any study that reported a single set to be superior to multiple sets [80].

However, the focus of this present review is not to evaluate the number of sets that provide the greatest strength or hypertrophy gains, but to investigate lower set volumes that can still provide significant benefits. Kraemer and Ratamess [80] in their review suggested that single sets would be more likely to be effective in the first 6–12 weeks of RT. A review paper in the Canadian Medical Association Journal recommended one to two sets of 8–12 repetitions for the novice training individual [14].

In summary, single sets have been shown to significantly and substantially increase muscle strength, especially in untrained individuals.

### Repetitions

The recommendation of 8–12 or 8–15 repetitions within a set for novice or previously untrained individuals is pervasive throughout the literature [14, 25, 50, 52, 71, 81–84]. The number of repetitions during a set interacts with the training load. Typically, the repetition endpoint (8–12 or 15 repetitions) would be determined by an inability to continue moving the specified resistance through a full range of motion, otherwise known as training to repetition failure. For resistance-trained men, Androulakis-Korakakis et al.’s [85] systematic review recommended that for suboptimal yet significant strength increases, a single set of 6–12 repetitions with loads ranging from approximately 70–85% 1RM reaching volitional or momentary (repetition) failure for 8–12 weeks can suffice. A meta-analysis of 13 studies ranging from 6 to 14 weeks’ training duration found that RT to repetition failure may provide similar or in some cases greater increases in dynamic strength and power, but no significant difference was detected for muscle hypertrophy when RT volumes were equalized [86]. A few reviews have concluded that training to repetition failure may provide greater strength [87], hypertrophy [88], and cardiorespiratory fitness [89] adaptations. Training to repetition failure did provide additional strength benefits in elite junior athletes [90], and may provide higher mechanical and metabolic stresses on the muscle [91, 92]. However, there are a number of studies that report no additional muscle strength [93–98] or hypertrophy [96] benefits of training to repetition failure. Conversely, an 8-week RT program reported greater enhancements in muscle strength, power, and rowing performance with repetition training not to failure compared to repetitions to failure in highly trained rowers [99].

For untrained individuals, the literature tends to indicate that one set of 8–15 repetitions will improve muscle strength and power without the need for lifting to momentary repetition failure or adding forced repetitions to increase the stress associated with more numerous repetitions. The number of repetitions to perform before reaching momentary failure may be detected by monitoring the movement velocity as the decrease in velocity is a strong indicator of the degree of fatigue before reaching momentary failure [100–102]. Kubo et al. [103] compared four, eight, and 12 repetition maximums with twice-weekly training over 10 weeks, and found that increases in muscle hypertrophy were similar among the three training protocols when the training volume was equated. However, they did report lower increases in muscle strength with the 12RM protocol.

Performing repetitions to momentary failure induces significantly higher ratings of perceived exertion [95, 100], which probably would not be pleasurable or motivating for individuals who are not highly motivated to exercise. Mangine et al. [104] used the term “repetitions in reserve” to describe ceasing repetitions prior to momentary failure. They found that the repetitions in reserve strategy allowed the workload to be maintained better across the sets at a lower rating of perceived effort.

Another strategy to increase repetitions and hence RT volumes is to incorporate advanced RT techniques such as “forced repetitions.” Forced repetitions involve a spotter providing some assistance to the lifter when momentary repetition failure is reached in order to allow the individual to force or add another few repetitions to the set volume. Wallace et al. [105] reported that whereas forced repetitions and other advanced RT techniques (e.g., drop sets, super sets, rest-pause sets) may induce small changes in volume load, muscle excitation, and fluid accumulation in strength-trained individuals, there were no significant differences in these variables compared to traditional RT when comparing single training sessions. Similarly, Drinkwater et al. [106] reported no additional strength benefits from forced repetitions with elite basketball and volleyball players (18–24 years) over a 6-week RT period. Other advanced RT routines such as drop sets and rest-pause sets can also decrease training time, but may be more effective for inducing greater hypertrophy than strength [38], while the increased intensity and discomfort would probably discourage the reluctant RT individual. Therefore, these advanced RT methods appear to be primarily suited for athletes accustomed to regularly performing RT.

A radical departure from the traditional recommendations was provided by Sato et al. [107] who had 13 young sedentary adults perform either a single 3-s isometric, concentric, or eccentric maximal voluntary contraction once a day for 20 days, while ten individuals were assigned to a control group. The single 3-s eccentric contraction group enhanced isometric (10.2 ± 6.4%), concentric (12.8 ± 9.6%), and eccentric maximal voluntary contraction (MVC) torque (12.2 ± 7.8%), with lower and less comprehensive increases for the concentric (isometric MVC torque: 6.3 ± 6.0%) and isometric training groups (eccentric MVC torque: 7.2 ± 4.4%). There were no muscle thickness changes in any group. Hence, with young sedentary adults, performing a single daily 3-s maximal voluntary contraction can increase muscle strength, with greater effects when using eccentric maximal voluntary contractions. It is likely that sedentary individuals performing single maximal contractions would soon accommodate this level of muscle stress decreasing the potential strength and power benefits, but it does illustrate how even such a minimal RT dose can initially (at least for 3 weeks) provide strength benefits. Future research should prolong the training period to determine the maximum training duration that elicits positive strength and power adaptations.

In summary, there is inconclusive evidence with regards to the number of repetitions needed to maximize physiological adaptations. While a large number of studies emphasize that repetition to failure is key to achieve optimal adaptations, other authors report similar adaptations with fewer repetitions. There is still a need for “unaccustomed stress” or suprathreshold stimuli to induce strength adaptations, but in the previously untrained, overcompensatory responses may still be achieved with repetitions near momentary failure but it may not be necessary to achieve full repetition failure. Differences in training status could be responsible for the controversial outcomes. In summary, individuals can employ 6–15 repetitions without extending themselves to repetition failure or using other advanced RT techniques such as forced repetitions.

## Resistance Training Intensity

In accord with previous research indicating that RT techniques that force the individual to continue to momentary (repetition) failure or even push past this point (e.g., forced repetitions, drop sets, rest-pause sets) may not be necessary to achieve significant and meaningful improvements in muscle strength, power, or hypertrophy, there is abundant research espousing the effectiveness of lower intensity RT. A meta-analysis by Rhea et al. [60] reported that RT with an intensity of 60% 1RM elicits maximal gains in untrained individuals, whereas 80% is most effective with trained individuals. Munoz-Martinez et al.’s [17] systematic meta-analytical review suggested that to elicit large effects in 1RM bench press, the RT intensity should be 30–60% 1RM, with sessions lasting at least 22.5–60 min. However, the low baseline fitness levels in this review might rationalize the lighter loads used in circuit training studies that exhibited higher strength gains. Sawan et al. [10] in their review suggested that RT health benefits can be attained by lifting lighter loads to volitional failure, and thus RT benefits may not necessitate heavier resistances. Higher intensity loads (80% 1RM) lifted three times per week for 6 weeks elicited greater neural adaptations than 30% 1RM but provided similar muscle hypertrophic responses as the lower intensity resistance [108]. Similar findings were reported﻿ by Schoenfeld et al. [109], who reported higher quadriceps and hamstrings activation with a leg press exercise at 75% 1RM versus 30% 1RM. In conclusion, the findings of a meta-analysis by Schoenfeld et al. [110] seem to summarize the overall findings. Whereas training with loads below 50% 1RM induces substantial strength and hypertrophy, improvements with untrained individuals, higher intensity or heavier loads tend to provide superior results. The evidence indicates that lower intensity RT programs can still provide substantial positive training adaptations in reluctant RT exercise participants.

## Type of Resistance Training

### Multi-Joint Versus Single-Joint Exercises

During multi-joint exercises such as cleans, snatches (Olympic type lifts), squats, deadlifts, or bench press, more muscles are involved [111–113] compared to single-joint exercises such as a biceps brachii curl [114]. Consequently, to train the main muscles involved in a deadlift (e.g., hamstrings, gluteal muscles, quadriceps, erector spinae) [112], four single-joint exercises would have to be performed to achieve similar results. One multi-joint exercise compared to various single-joint exercises (with the same set and repetition range compared to the multi-joint exercise) can increase muscle strength and power to an even greater extent [38, 115–117]. With total work volume equated, a multi-joint RT group demonstrated greater gains than a single-joint RT program group in bench press, knee extension, and squat 1RMs, suggesting multi-joint exercises were more efficient for improving muscle strength [115]. Hoffman et al. [118] noted an 18% greater improvement in squat 1RM and a twofold better improvement in 40-yard sprint time with the more complex multi-joint Olympic lifts versus powerlifting. Similarly, Channel and Barfield [119] reported that multi-joint Olympic lifts provided a modest advantage over powerlifting for vertical jump height improvement in high school athletes.

Consequently, it is suggested that multi-joint exercises can be considered as more time efficient by reducing the overall number of exercises (i.e., less training time with similar results) [38, 120]. In addition, multi-joint exercises stimulate interlimb coordination, which is not the case with single-joint exercises. Furthermore, activities of daily living are typically performed in a standing position requiring some level of core strength and stability and thus these multi-articular exercises (i.e., squats, deadlifts and others) that mimic everyday tasks [115] should be incorporated [121, 122]. Accordingly, there is no need to add single-joint exercises to multi-joint exercises within a RT session, since the additional effects are negligible [123, 124] and the inclusion of specific exercises to activate individual stabilizer muscles (e.g., transversus abdominis, internal obliques) has not been adequately justified [125]. Similar principles apply to older adults for whom multimodal or combined exercise interventions that include balance, perturbation, and dual-task exercises are recommended [126].

Both similar [127] and higher [128–130] muscle activation have been reported with free weight multi-joint exercises compared to machines while injury prevalence seems to be higher with free weights compared to machines, especially for inexperienced individuals [131]. This increased injury incidence is likely due to improper technique and the higher degrees of uncontrolled movements (i.e., stabilizing the trajectory of the external load). Consequently, guided machines (e.g., Smith machine) can minimize the prevalence of such injuries [131]. Hence, it can be suggested that if the technique of the multi-joint exercises is not yet fully developed or coordinated, training with guided machines can be an initial suitable substitute for such a peer group. Training with free weights under the supervision of a certified trainer would also be an appropriate recommendation to avoid injuries and optimize performance gains.

A multi-joint exercise approach is not only valid for an untrained or sedentary person but also for time-limited endurance athletes, such as triathletes who have such high endurance training loads, in terms of recovery, as these athletes may not perform additional comprehensive RT. Beneficial effects of RT for endurance performance as well as endurance economy as an addition to the typical endurance training were reported [132–134]. Thus, multi-joint exercises such as squats, deadlifts, bench press twice a week [133] may be enough to enhance endurance performance/economy with RT.

### Functional Training

La Scala Teixeira et al. [117] defined functionality as an approach to stimulate different physical abilities in an integrative manner to improve performance in daily activities. Despite individual differences, some actions apply to almost everyone, such as walking, pushing, and pulling. In most of these everyday activities, we concomitantly use physical fitness in the form of muscle strength, flexibility, coordination, and others, as shown in the physical activity recommendations [30, 135]. A suitable option for the functional approach is to explore the complexity of the exercises (e.g., biomechanical variations) to modify the stimulus intensity [116]. Thus, if the primary purpose of the training program is to improve function, it will be necessary to integrate different physical abilities and apply the specificity principle [136] to get the maximum benefit.

Muscle power and strength are essential to preserve physical function [137]; however, muscle power shows larger declines with ageing compared with muscle strength [138]. Hence, power training (i.e., maximum intended velocity during the shortening of the muscle) [139], which typically utilize lower loads (e.g., 60–80% of 1RM) [140], could be a helpful approach to maintain or even improve physical function [136], especially in older adults. As mentioned previously for strength training (see Sect. 5.1), these power movements may be initially safer (decreased chance of injuries) with machine resistance or supervised free-weight exercises.

Principal movement pattern exercises can be combined in a circuit format or supersets (performing two exercises consecutively without rest or very short rest) to make the training more dynamic, reduce the session duration, maintain the benefits with lighter loads as well as improve cardiorespiratory fitness [17]. Individual preference and tolerance to exercise will define whether circuit training, supersets, high-intensity interval training (HIIT), or other advanced techniques are incorporated into the training.

## Stretch Training as an Alternative to Resistance Training

Although RT is the most common method to increase muscle strength and hypertrophy [46, 141, 142], the reluctant exerciser may not be inclined to participate in exercises they perceive to be too intense, painful, and uncomfortable or they may not have access to the required equipment or fitness facility. Although not time efficient, recent research illustrates that prolonged static stretching can produce muscle strength and hypertrophy benefits [143–145] with minimal intensity of effort (vs. minimal time concerns).

Boppart and Mahmassani [146] and Aguillar-Agon et al. [147] pointed out that mechanical tension was a sufficient stimulus to induce physiological adaptations such as increased protein turnover [148–152]. Animal model stretch-training studies reported significant large magnitude muscle mass increases [153] using comparatively high stretching durations of 30 min [154, 155] to 24 h daily [153, 156, 157]. However, since stretching induced mechanical tension without the need for active movement or even innervation of the muscle [158, 159], the question arises about the transferability to humans.

With stretching, there is little required equipment and thus it can easily be integrated into human daily activity. However, Nunes et al. [160] reviewed the current literature showing no significant stretch-mediated hypertrophy in humans. It is noteworthy that stretching duration did not extend past 2 min per session and was mostly performed 2–3 days per week. Other studies were also not able to induce significant hypertrophy in response to 4–6 weeks of stretching, using stretching durations of 360 s (1 × 360 s vs. 3 × 120 s per week) [161], 4 × 30 s [162] to 6 × 5 min [163] 2–3 days per week. In contrast, the participants in the study by Simpson et al. [164] stretched the plantar flexors for 3 min, five days per week for 6 weeks, demonstrating a significant 5.6% increase in muscle thickness.

Warneke et al. [165] conducted long duration stretching studies in humans, stretching the plantar flexors for up to two continuous hours per day using an orthotic stretching device [166, 167], and found significant, large magnitude increases in muscle strength (22.9%, *d* = 0.91) and hypertrophy (15.2%, *d* = 0.84). The authors highlighted the possibility of using the stretching device while watching television, playing computer games or doing work in a sitting position [166, 167]. However, there seems to be substantial limitations of this training method, since stretching durations of up to 120 continuous minutes per day per muscle would be difficult to sustain [168] and it can be assumed that most people would not be able or willing to perform a weekly stretching volume of 420–840 min per week per muscle group [166]. Subsequently, this research group then compared daily 1-h plantar flexors stretching versus a commonly used RT exercise using calf raises 3 days per week with 5 × 12 repetitions per session. Both the stretching and RT interventions significantly increased maximum strength, flexibility, and muscle thickness to similar extents. Furthermore, the authors tested the effectiveness of 10 minutes’ daily stretching using a stretching board, and were still able to induce significant increases in muscle strength [165]. The literature investigating stretch-mediated (≥ 30 min per session) hypertrophy is very scarce and has exclusively tested the plantar flexors [163, 166, 167, 169, 170]. More research is necessary to address the optimal dose–response relationship with more muscles and diverse participants. Recently, a meta-analysis of 41 chronic stretching studies by Arntz et al. [145] calculated trivial to small magnitude increases in muscle strength and power (median weekly session frequency was three (range 2–14), with a median intervention period of 6 weeks (range 2–24)). Subgroup analyses showed larger strength gains in female, older, and sedentary individuals. A greater number of stretch repetitions (mean stretching time per exercise was 30 s with a range of 2–300 s) provided larger muscle strength improvements.

If time efficiency is the major issue limiting training participation, then the aforementioned static stretch training programs would certainly not be favourable. However, for those individuals with abundant time, but who are not enthusiastic about moderate- to high-intensity RT, prolonged passive static stretching while seated at work or watching television or the computer at home might be another alternative for improving muscle strength and hypertrophy (minimal intensity rather than minimal time).

## Conclusion and Recommendations

There are volumes of research on optimal RT programs to achieve maximal strength, power, and hypertrophy. A meta-analysis by Peterson et al. [79] identified the optimal training parameters for three distinct groups. Maximal strength gains for untrained individuals are elicited at a mean training intensity of 60% of 1RM, 3 days per week, and with four sets per muscle group. Recreationally trained individuals can achieve maximal strength gains with 80% 1RM, 2 days per week, with a volume of four sets. For athletes, 85% of 1RM, 2 days per week, and with eight sets per muscle group, would be optimal to achieve maximal strength gains. While this research highlights the optimal prescriptions for maximal strength gains for the more motivated, or aspiring fitness enthusiasts or athletes, there is a significant portion of the population that are resistant or averse to RT and uninterested in “maximal” strength gains. Since RT can promote positive health adaptations for a myriad of health conditions, it is imperative to find a means to motivate those unwilling or challenged to begin RT.

It is not presently known whether such minimalist RT programs can positively impact the overall health of an individual. However, 2–3 months of a minimalist program has the potential to improve muscle strength in individuals with little RT experience. These recommendations are in accord with a systematic review by Androulakis-Korakakis et al. [85], who suggested that just a single set of 6–12 repetitions at approximately 70–85% 1RM, 2–3 times per week with high intensity of effort for 8–12 weeks can produce significant increases in squat and bench press 1RM strength in resistance-trained men. Consequently, these positive adaptations with lower volumes of RT could introduce and motivate the individual to continue with greater training dosages (RT frequencies, volumes, and intensities) that have been associated with many health benefits.

An advantage for those individuals who previously performed RT and were now detrained is that the initial return to training may provide more rapid training adaptations (i.e., muscle or neuromuscular memory). A foundation of prior RT would have established neural adaptations (e.g., increased motoneuron recruitment, rate coding, and synchronization) that would be more easily retained than with individuals who had never previously trained [3, 171]. Furthermore, due to the prior activation of satellite cells and the infusion of myonuclei, previous RT individuals would have a greater capacity for protein synthesis facilitating more rapid training adaptations [172, 173]. One would expect that the combination of more rapid neural and morphological adaptations contributing to increased muscle strength and hypertrophy would help to maintain the motivation to continue RT.

Furthermore, RT adaptations are reported to be global with unilateral training of one limb promoting strength adaptations in contralateral homologous and heterologous muscle groups (cross-education) [174–177]. These global responses are also evident with stretching [178, 179] and foam rolling [180]. Thus, it is not necessary to spend an inordinate amount of time resistance training or stretching all individual muscle groups as there will be global effects on non-exercised muscles.

RT-hesitant individuals who are only interested in a minimalist program should begin with a single RT session per week for at least 8–12 weeks. This weekly session should consist of at least one set of 6–15 repetitions ranging from 30 to 80% of 1RM using multi-joint movements/exercises that emphasize functional movements of daily living (i.e., pushing, pulling, and walking). It is also recommended to perform the concentric action as fast as possible to promote power. Progression in terms of exercise volume and intensity should be realized over the training period. For those who seek even lower intensity strength and hypertrophy enhancing exercises, prolonged static stretching may be an alternative for some muscle groups. While much of this research is based on RT programs of 8–12 weeks, it is not known if these parameters would still be effective for substantially longer durations. It may be necessary after three or more months of training to provide greater training stimuli with increases in weekly frequency of training (e.g., two or more session per week), volumes (e.g., multiple sets), intensities, and exercise types (add power training such as plyometrics or metastable/unstable environments).
